# Supplementary material for: Multiple golgins are required to support extracellular matrix secretion, modification, and assembly
Source: J Cell Biol. 2025 Aug 18;224(10):e202411167. doi: 10.1083/jcb.202411167 (PMC12360289; doi:10.1083/jcb.202411167)

Fig. 2a media and lysate - collagen type 1

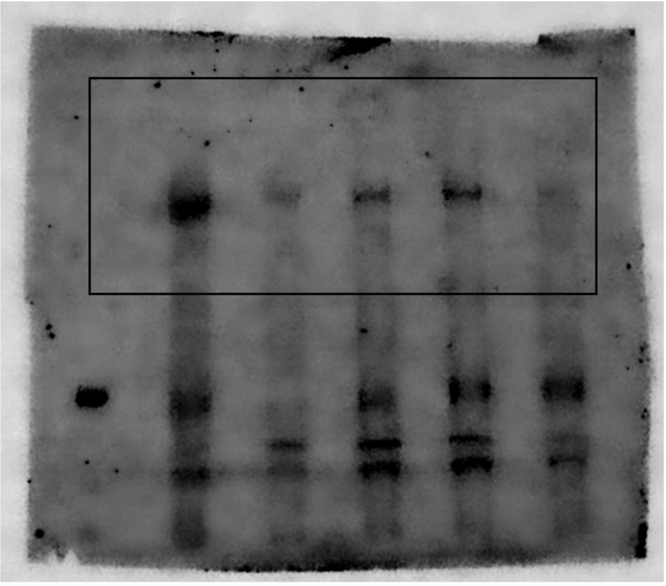

Total Protein stain

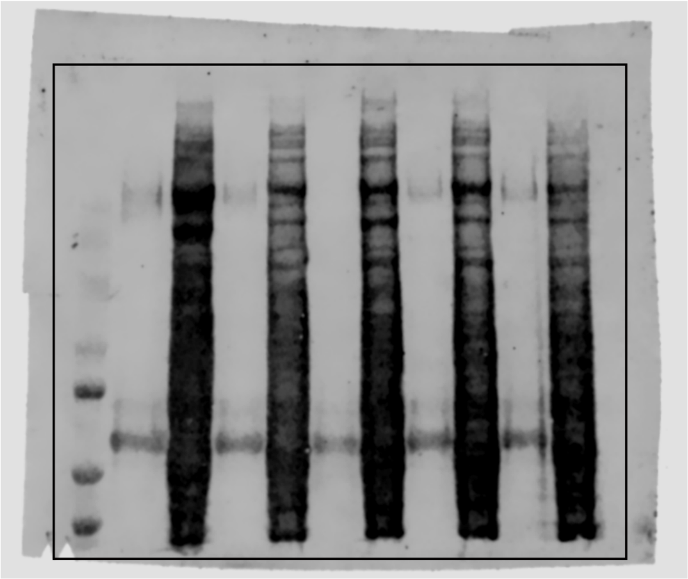

Fig. 2b matrix - collagen type 1

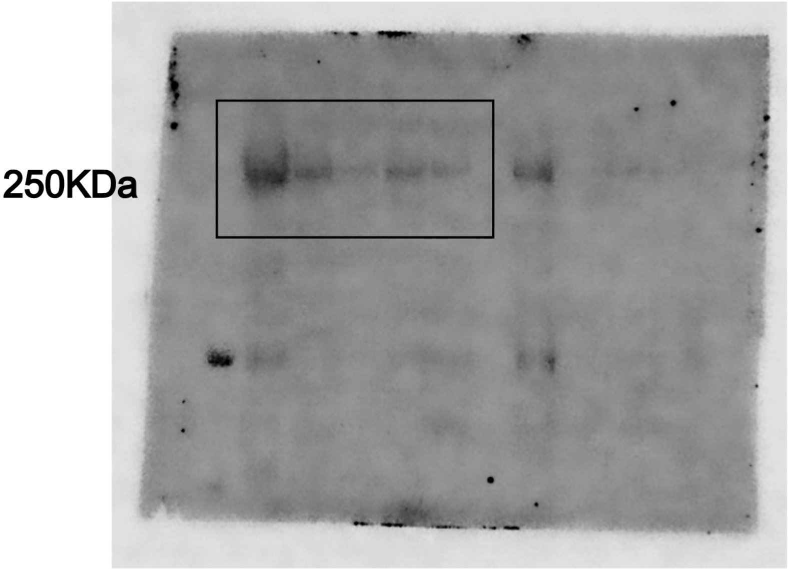

Total Protein stain

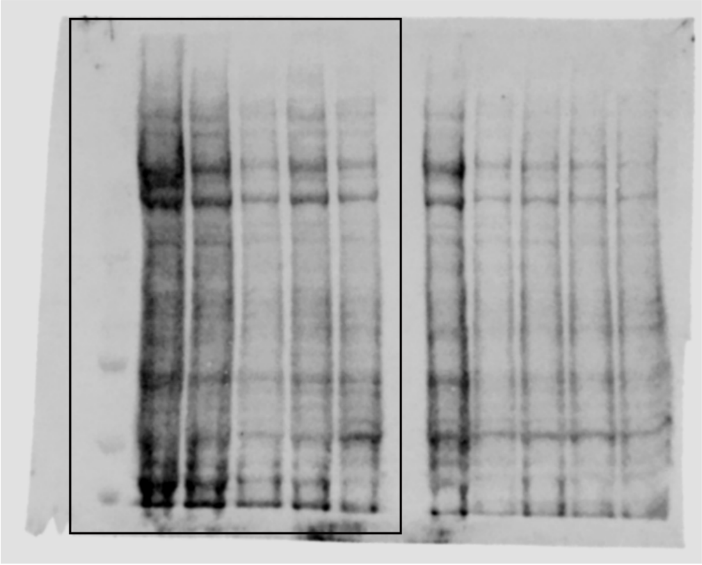

Supplement: SourceData F2 — is the source file for Fig. 2. [file jcb_202411167_sourcedataf2.pdf]
