# Supplementary figures and images for: Multiple golgins are required to support extracellular matrix secretion, modification, and assembly
Source: J Cell Biol. 2025 Aug 18;224(10):e202411167. doi: 10.1083/jcb.202411167 (PMC12360289; doi:10.1083/jcb.202411167)

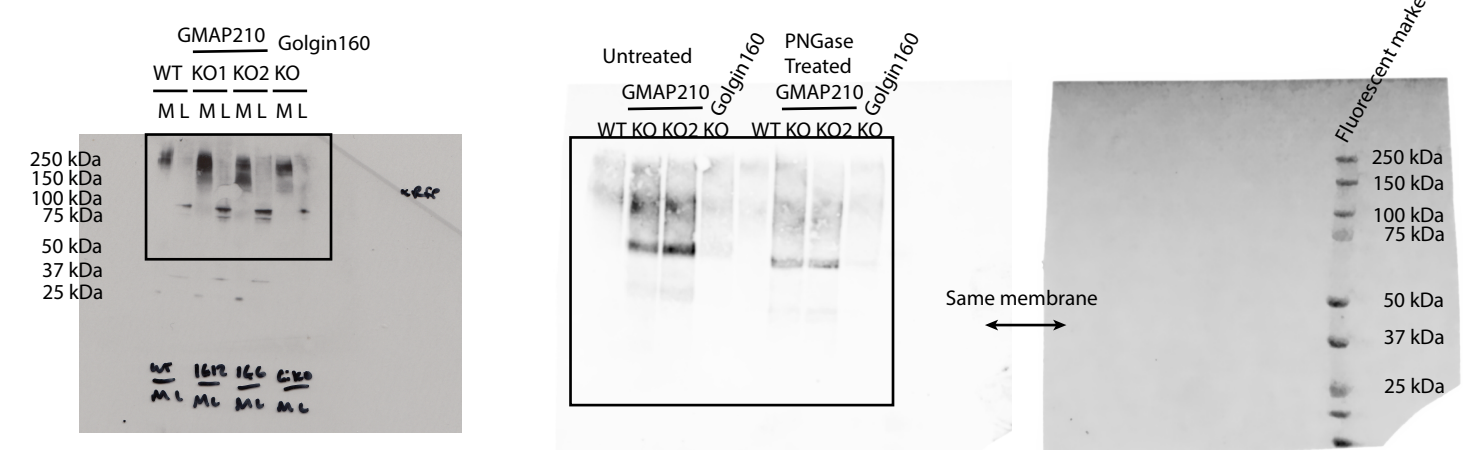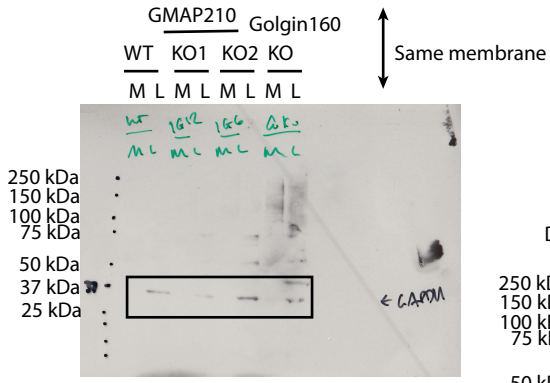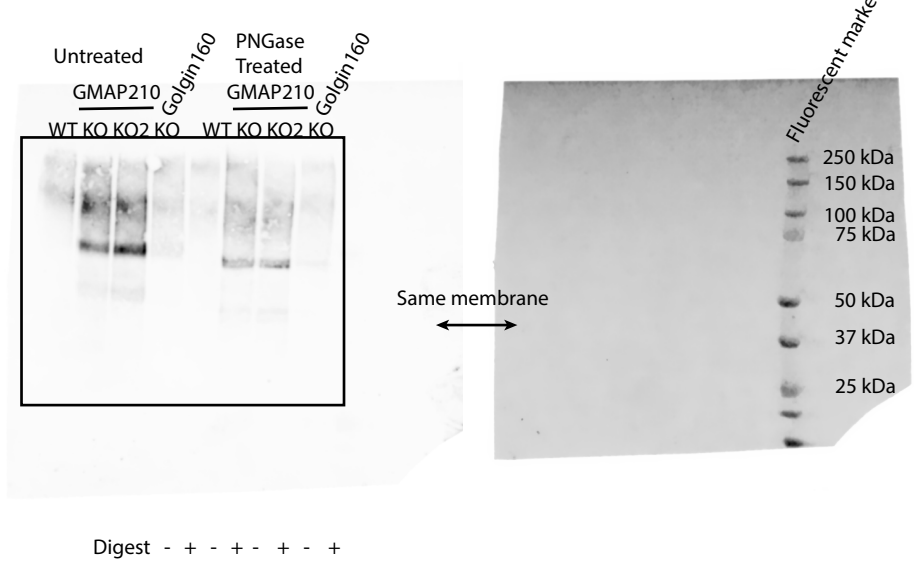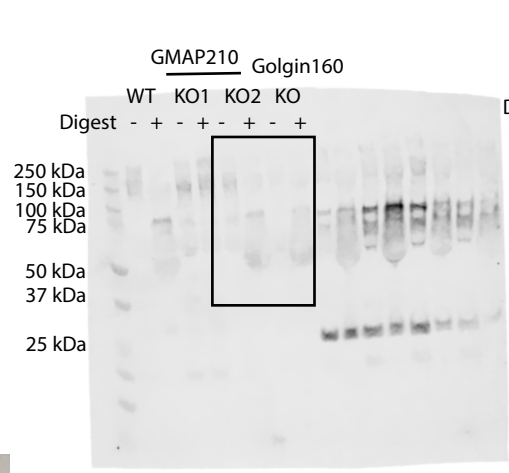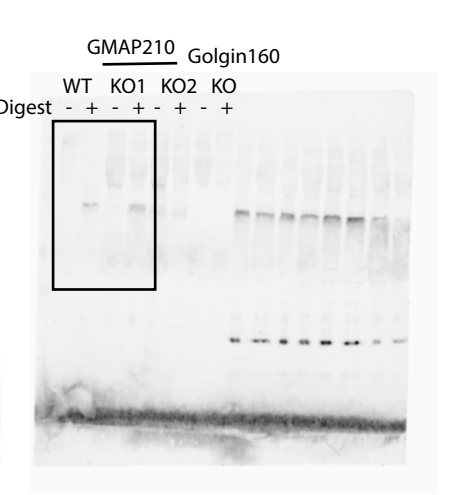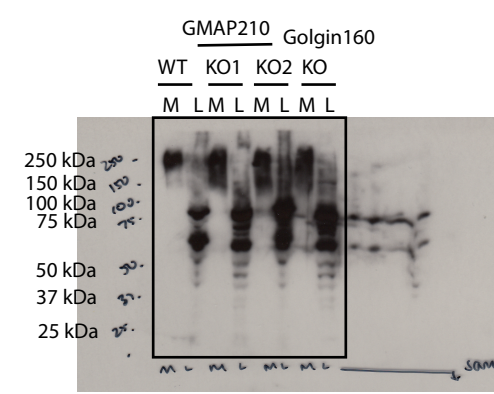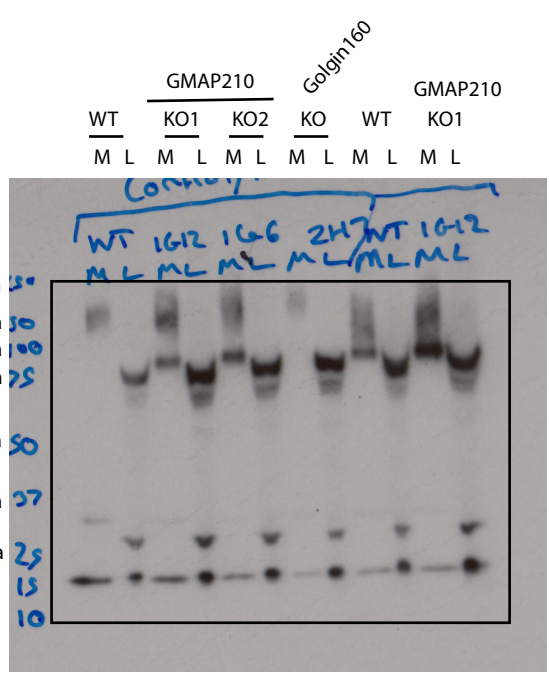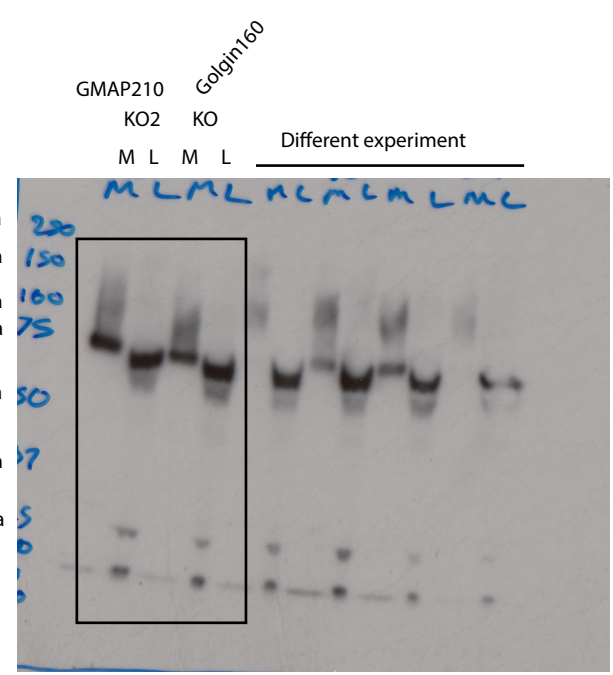

Supplement: SourceData F8 — is the source file for Fig. 8. [file jcb_202411167_sourcedataf8.pdf]

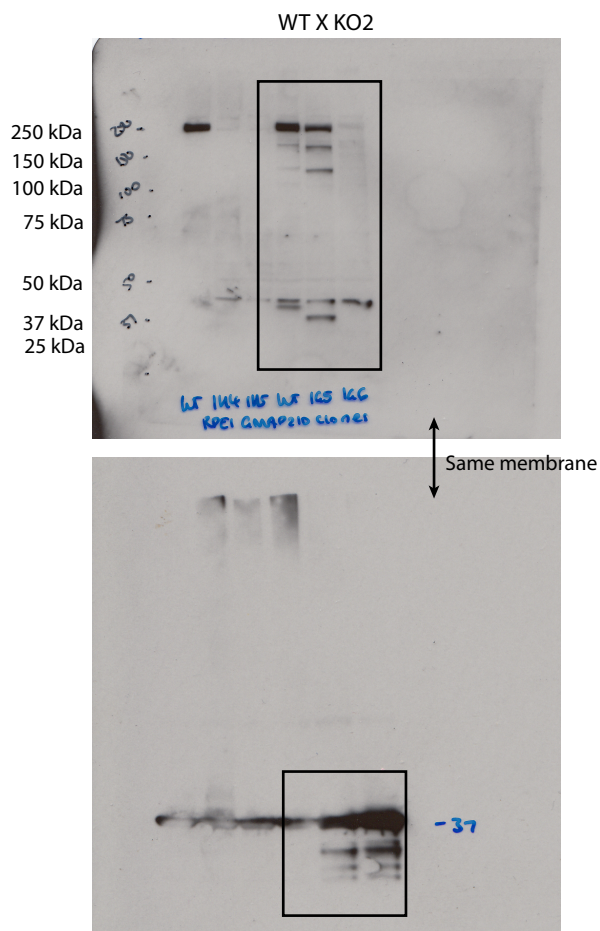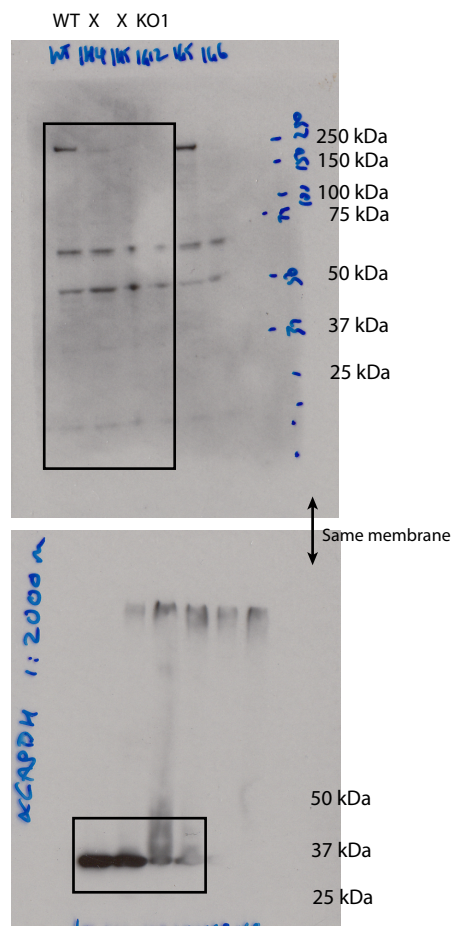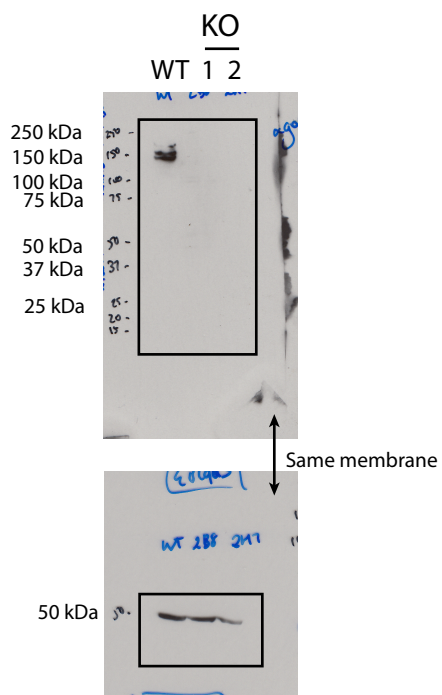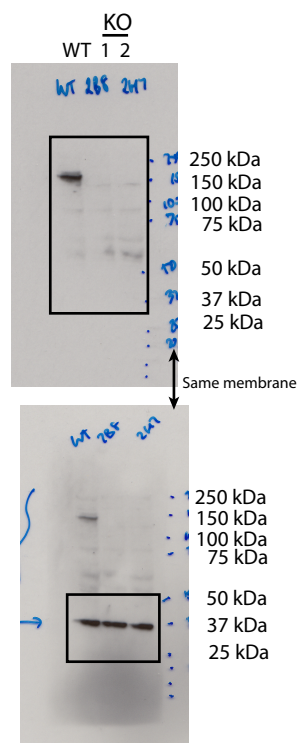

Supplement: SourceData FS1 — is the source file for Fig. S1. [file jcb_202411167_sourcedatafs1.pdf]

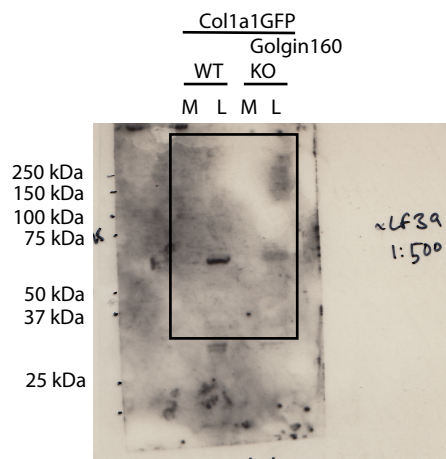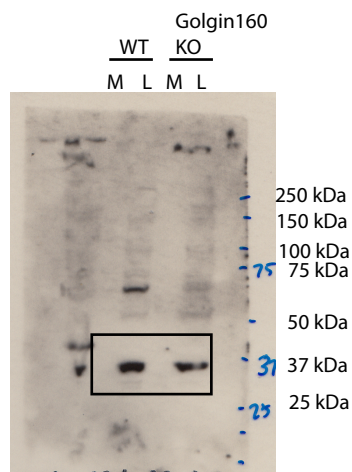

Supplement: SourceData FS2 — is the source file for Fig. S2. [file jcb_202411167_sourcedatafs2.pdf]
